# Degree and centrality-based approaches in network-based variable selection: Insights from the Singapore Longitudinal Aging Study

**DOI:** 10.1371/journal.pone.0219186

**Published:** 2019-07-18

**Authors:** Jesus Felix Bayta Valenzuela, Christopher Monterola, Victor Joo Chuan Tong, Tamàs Fülöp, Tze Pin Ng, Anis Larbi

**Affiliations:** 1 Computing Science Department, Institute of High Performance Computing, Singapore, Singapore; 2 Analytics, Computing and Complex Systems Laboratory, Asian Institute of Management, Makati City, Philippines; 3 Aboitiz School of Innovation, Technology and Entrepreneurship, Asian Institute of Management, Makati City, Philippines; 4 Social and Cognitive Computing Department, Institute of High Performance Computing, Singapore, Singapore; 5 Yong Loo Lin School of Medicine, Department of Biochemistry, National University of Singapore, Singapore, Singapore; 6 Department of Medicine, University of Sherbrooke, Quebec, Canada; 7 Yong Loo Lin School of Medicine, National University of Singapore, Department of Psychological Medicine, Singapore, Singapore; 8 Singapore Immunology Network, Singapore, Singapore; 9 Yong Loo Lin School of Medicine, National University of Singapore, Department of Microbiology and Immunology, Singapore, Singapore; 10 School of Biological Sciences, Nanyang Technological University (NTU), Singapore, Singapore; 11 Department of Biology, Faculty of Sciences, Tunis El Manar University, Tunis, Tunisia; Universidad Rey Juan Carlos, SPAIN

## Abstract

We describe a network-based method to obtain a subset of representative variables from clinical data of subjects of the second Singapore Longitudinal Aging Study (SLAS-2), while preserving to a good extent the predictive performance of the full set with regards to a multi-faceted index of successful aging, *SAGE*. To examine differences in predictive performance of high-degree nodes (“hubs”) and high-centrality ones (“cores”), we implement four subsetting strategies (two degree-based, two centrality-based) and obtain four surrogate sets of variables, which we use as input features for machine learning models to predict the *SAGE* index of subjects. All four models have variables belonging to the physical, cardiovascular, cognitive and immunological domains among their fifteen most important predictors. A fifth domain (leisure-time activities, *LTA*) is also present in some form. From a comparison of the surrogate sets’ size and predictive performance, a centrality-based approach (selection of the most central variable-nodes within each cluster) yielded the smallest-sized surrogate set, while having high prediction accuracy (measured by its model’s area-under-curve, AUC) in comparison to its analogous degree-based strategy (selection of the highest-degree nodes per cluster). Inclusion of the next most-central variables yielded negligible changes in predictive performance while more than doubling the surrogate set size. The centrality-based approach thus yields a surrogate set which offers a good balance between number of variables and prediction performance, and can act as a representative subset of the SLAS-2 clinical dataset.

## Introduction

Population aging has implications for society, the economy and policy-making. A report released by the United Nation’s Population Division [1] showed that the global share of older people (defined as those above the age of 60) stood at 11.7% in 2013, and is projected to reach 21.1% by 2050, with one-third of the 2013 global share residing in developed countries. The increasing number of aging people is putting pressure on countries which already have low old-age support ratios (the number of working-age adults per elderly person in a given population), and presents a host of demographic, economic and socio-cultural challenges.

Thus, research into human aging has attracted significant interest from a broad spectrum of institutions and disciplines. A significant portion of this endeavour, from the clinical perspective, consists of identifying a minimal set of clinical variables predictive of clinical trajectories in aging. Such a minimal dataset would represent substantial savings, in terms of costs, effort and time required to gather the necessary information. In addition, this ultimately will enable the stratification of populations at risk for various age-related diseases.

One instance where a minimal dataset can help is in the operationalization of successful aging, something which has not met with consensus, with several competing definitions present in the literature [2]. Rowe and Kahn’s [3, 4] three-factor model distinguishing between “usual” and “successful” aging proved highly influential, but also led to a vigorous debate and a number of critiques, among which are those who proposed additional factors spanning a broader range of domains (such as [5, 6]) or rejected Rowe and Kahn’s conception entirely [7]. Conceptual differences between researcher-driven operational definitions and lay-based, qualitative perspectives have also been found [8].

In another instance, frailty assessment is currently performed by several competing operational definitions of frailty [9–11]. Fried’s [12] phenotype-based definition, which approaches frailty from the standpoint of a decline in physical function, uses a fixed set of five criteria (shrinkage, exhaustion, weakness, low activity and slowness). The second one, Rockwood and Mitnitski’s Frailty Index (FI) [13] treats frailty as the accumulation of deficits across domains (including physical and cognitive function as well as physiological measures). This index uses a large set consisting of various clinical conditions and diseases. A third index, the Tillburg Frailty Indicator [14], also envisions a multi-domain approach to frailty, but relies on a set of domain-related questions. Of the three, Fried’s criteria is the most commonly-used due to the small size of its required dataset; the other two, while covering other domains aside from the physical, require more data to be collected. While frailty is one among many age-associated syndromes, there is a need to better master data generated from large cohort studies in order to cover all diseases and syndromes.

Both instances can be considered as belonging to the more general problem of feature selection in bio-informatics [15–17]. Two tripartite conceptual frameworks have been proposed to categorize feature selection methods: Saeys *et al*.’s filter / wrapper / embedded taxonomy [15] and the more recent supervised / semi-supervised / unsupervised classification [17]. Regardless of schema, most feature selection methods used in bioinformatics are of the supervised / wrapper / embedded type, where information from the outcome variable directly or indirectly contributes to which feature variables are ultimately selected. Such methods run the risk of over-fitting and poor generalization, and thus sub-optimal results when used on new or independent data.

In this paper we examine several different network-based strategies for the reduction of variables, in terms of their predictive performance for *SAGE* a multi-domain index of successful aging. Network analysis has previously been used to identify hubs associated with human brain function and disorders [18–23]. However, the usefulness of these hubs as a proxy subset for the larger dataset (and thus, a means of dimensionality reduction) is something which has not received sufficient attention from the existing literature. The current study is based on the clinical phenotype data from the Singapore Longitudinal Aging Study (SLAS-2). We propose to construct a pairwise effect-size network from the input data, and obtain from it clusters consisting of tightly-associated variables. We subsequently apply four subsetting strategies (two based on the node degree and two on the betweenness centrality) to obtain subsets of variables (“surrogate sets”), which we use to train classifier models to predict *SAGE*. Finally we compare the performance of these models (and one trained on all dataset variables) using *AUC*, the area under each model’s receiver-operating-characteristic (ROC) curves. Motivated by the need to identify a smaller subset of practically-measurable key variables, we forgo using combinations or transformations of variables in data reduction.

## Materials and methods

### The SLAS-2 dataset

We examined data from the second cohort of the Singapore Longitudinal Aging Study (hereafter referred to as SLAS-2). The study participants consisted of 3270 elderly residents of Singapore’s south-central and southwest regions. The SLAS study, and its methods, have been described previously [5, 24, 25]. Briefly, from 2010 to 2013, all residents of the aforementioned regions aged 55 and above were identified from a door-to-door census, and were invited to participate in the research. The participants were aged 55 and above when baseline surveys for SLAS-2 were conducted. All participants who were able, provided informed consent in writing prior to obtaining their data. In instances where cognitively-impaired participants were unable to provide informed consent (such as cases where severe physical or cognitive impairment due to debilitating or terminal illness or dementia were involved), their closest adult next-of-kin provided the informed consent. Approval of this study, including the inclusion of physically or cognitively impaired individuals following the consent of adult next-of-kin, was granted by the Institutional Review Board of the National University of Singapore (**NUS-IRB 04-140**).

For each participant, physical, metabolic, respiratory and serological measurements were taken, along with several surveys covering the subjects’ status across several fields, such as physical (e.g. the Activites of Daily Living (ADL) survey), cognitive (e.g. the Mini Mental State Examination (MMSE)), and emotional (e.g. the Geriatric Depression Scale (GDS) survey). Medical histories as well as dietary habits of the subjects were also obtained. All told, we identified 1579 usable variables (683 numerical and 896 categorical), which is further reduced to 1373 (664 numerical and 709 categorical). The process of reduction is described in the next section.

### Statistical analyses

We form all possible unique pairs of the 1579 variables, and calculate effect-sizes for each pair. Depending on the type of variables in a pair (both numerical or categorical, or mixed), we used the measures listed in Table 1. We use Bergsma’s finite-sample correction for the Cramer’s *ϕ*, as it exhibits a large bias otherwise [26].

**Table 1:** Effect-size measures and significance tests for pairwise analysis, according to the type of variables in each pair.

Pair Composition	Statistical Test	Effect Size Measure
Both numerical	Student’s *t* test on Spearman’s *r*	Spearman’s *r*^2^
Both categorical	*χ*^2^ test on contingency table	Cramer’s *ϕ*^2^
One numerical, one categorical	Kruskal-Wallis ANOVA + Dunn’s *post hoc* test	$Z_{max}^{2}/n$

Table 1 also gives the corresponding tests for statistical significance we use for each pairwise effect-size. We use a significance level of 0.05, and apply the Bonferroni correction for multiple comparisons. All statistical tests we used are nonparametric, so as to have minimum assumptions regarding the distribution of the data. Regarding Dunn’s *post hoc* test used when the Kruskal-Wallis analysis of variance [27] gives a significant result, we use $Z_{max}^{2}/n$ as the effect-size, where *Z*_*max*_ is the maximum *Z*-score returned by Dunn’s test, and *n* is the number of samples used in the analysis of variance. All calculations were performed in Python using the SciPy library [28], except for the Kruskal-Wallis and Dunn’s tests, which used the Python implementation by Muldal [29].

**Fig 1:**
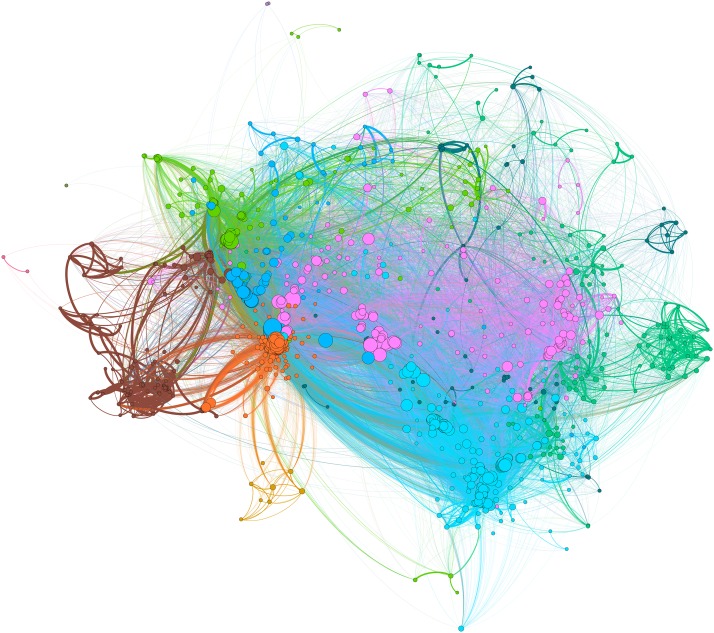
Giant component of a pairwise effect-size network constructed from SLAS-2 data (N = 1373, E = 101030). Graph visualization is done in Gephi [30].

From the statistically-significant pairs of variables, we construct a pairwise effect-size network, in which the *nodes* represent the variables we used, and *edges* between nodes represent statistically-significant associations. The pairwise effect sizes serve as the edge *weights*. The network we thus obtain is a disconnected network, consisting of one large subnetwork of *N* = 1373 variables (the *giant component*), a pair of correlated variables (*w0_total1* and *w0_total2*, the total energy expenditure of a subject during weekdays and weekends respectively, with *R*^2^ = 0.17) unconnected to anything else, and the rest of the 1579 variables wholly disconnected (*singleton* nodes). We focus on the giant component in this work, as it comprises the bulk of the network. Fig 1 shows this giant component network (*N* = 1373, *E* = 101030).

### Network and clustering

From the giant network, we construct minimum spanning trees (MSTs). Given a connected and weighted network **G** with *N* nodes and *E* weighted edges, the MST of **G** is an acyclic subnetwork having the same nodes as it, such that the sum of all edges is at a minimum. It has been shown to be useful in clustering bioinformatics information such as microarray data [31, 32], and has emerged as a widely-used tool in phylogenetic and molecular epidemiological analyses [33–37]. For the weight, we use a distance transformation of the effect size (which we call the *effect-size distance*) as follows. Given an effect-size measure *R*^2^, the transformation into the effect-size distance *d* is given by Eq 1:

$$\begin{array}{c} d=\frac{1-R^{2}}{R^{2}} \end{array}$$ (1)

**Fig 2:**
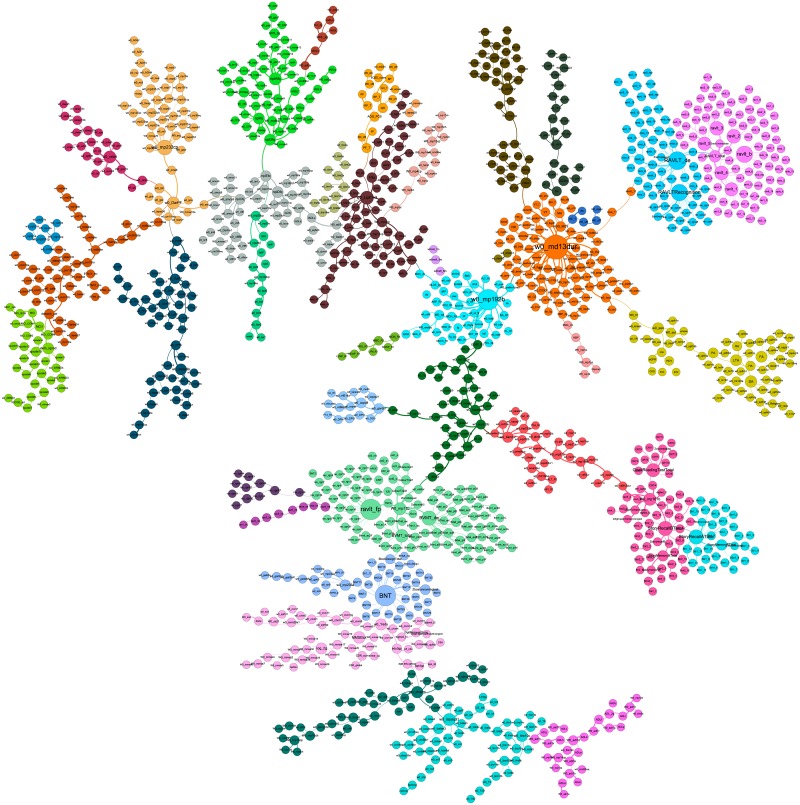
A minimum spanning tree (MST) of the giant component network shown in Fig 1. The MST is obtained using Kruskal’s algorithm.

The MST thus gives a “backbone” of the network consisting of the strongest variable associations, as *d* and *R*^2^ are inversely-proportional. Fig 2 shows an MST of the network obtained by Kruskal’s algorithm [27], as implemented in Python’s NetworkX library [38].

The uniqueness of an MST for a network is only mathematically guaranteed if the network’s edge weights are distinct. Furthermore, Kruskal’s algorithm, which works by ordering network edges according to increasing distance, is susceptible to the order by which edges having the same distance are enumerated. Thus we generate multiple MSTs by slightly-modifying Kruskal’s algorithm: within the sequence containing the edges arranged with increasing distance, we shuffle the ordering of any edges with equal distances, and obtain an MST for each shuffling. We obtained 250 MSTs using this method.

**Fig 3:**
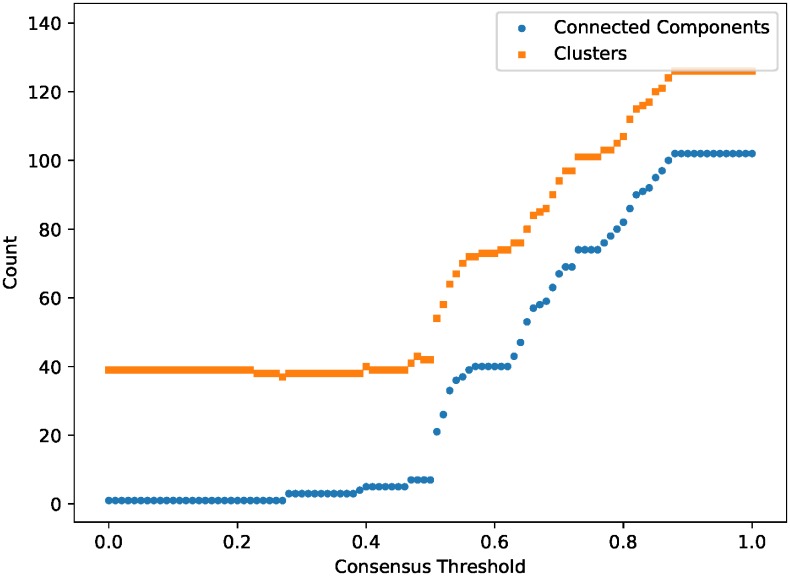
Threshold fraction, number of connected components and clusters for a consensus network of 250 minimum spanning trees obtained from pairwise effect-size analysis of SLAS-2 data. Clustering was performed on the consensus network using Blondel’s algorithm [39].

There are multiple ways to combine the results of the obtained MSTs into a single network. Among them are *consensus* rules, in which an edge present in a fraction of the trees exceeding a set threshold is retained by the consensus network. Fig 3 shows the number of connected components a consensus network has varying with the threshold used. We note that the consensus network is connected up to the threshold value of 0.27, above which it splits into three connected components, with minor increases up to 0.50 (seven connected components). Above 0.50, the number of network pieces jumps to 21 and begins to increase sharply, reaching 102 components at 0.88 and stabilizing.

We chose to combine the results represented by these MSTs into a majority-rule consensus network, corresponding to a threshold of 0.5. The majority rule criterion is in common use to summarize multiple MSTs of a single network [40], and has been shown to have a justification from a Bayesian standpoint [41]. We retain all edges satisfying the criterion to obtain the consensus network.

After obtaining the consensus network from the MSTs, we cluster the nodes using Blondel’s algorithm [39]. This algorithm partitions a network such that the *modularity* [42], a measure of the strength of the connections among nodes within the same cluster, and simultaneously the weakness of connections of nodes belonging to different clusters, is maximized. The modularity ranges from -1 to 1, with positive values corresponding to stronger intra-cluster links. It is given by Eq 2:

$$\begin{array}{c} Q=\frac{1}{2m}\sum_{i}\sum_{j}[A_{ij}-\frac{k_{i}k_{j}}{2m}]\delta_{c_{i},c_{j}} \end{array}$$ (2)

where *Q* is the modularity, *A*_*ij*_ is the weight of the edge connecting nodes *i* and *j* (zero if the two are unconnected), $m=\frac{1}{2}\sum_{i}\sum_{j}A_{ij}$ is the sum of all the edge weights in the network, *k*_*i*_ and *k*_*j*_ are the sum of the weights of edges connected to *i* and *j*, respectively, *c*_*i*_ and *c*_*j*_ are labels for the clusters *i* and *j* belong to, and *δ* is the Krönecker delta. Here we use the original calculated effect-size measure *R*^2^ as the edge weight, instead of the effect-size distance *d*. Fig 3 also shows how the number of clusters determined this way vary with the threshold used to construct the consensus network. We have previously seen that 0.5 marks the threshold above which the consensus network drastically fragments into many small components, and we find the same behavior for the number of clusters detected, generally remaining stable around 38 clusters, before sharply rising above the 0.5 threshold, saturating at 126 clusters at 0.88, the same as with the number of connected components. At the majority-rule criterion threshold, we obtain 42 clusters via Blondel’s algorithm.

**Fig 4:**
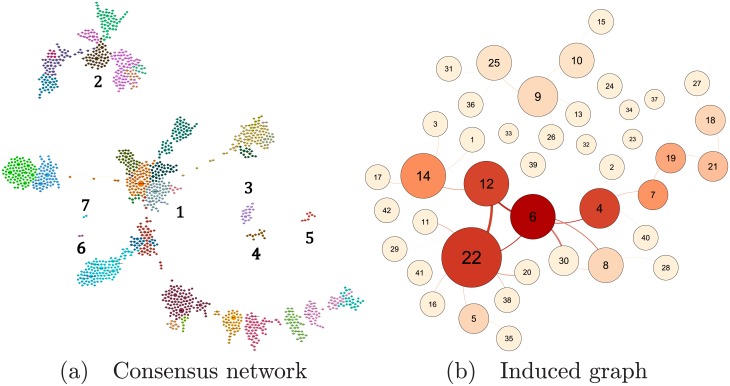
(a) Majority-rule consensus network and (b) its induced graph obtained from 250 MSTs of the giant component network in Fig 1, showing the clustering obtained by Blondel’s algorithm [39]. Different colors correspond to different clusters in (a), and different betweenness centrality of the clusters in (b). The clusters in (a) are labeled as a visual aid. In both, node sizes represent node degree (number of nearest neighbor nodes): variables in (a) and clusters in (b).

Fig 4a shows the clustering pattern of the consensus network, where nodes belonging to a given cluster colored identically. Fig 4b shows the corresponding induced graph of the consensus network. The induced graph of a network is a schematic representation of the latter, where the nodes are the clusters of the original network, and edges represent adjacency of the clusters: connected induced-graph nodes correspond to connected clusters in the original under the majority-rule criterion.

### Variable selection and surrogate sets

To achieve reduction of the number of variables required, from each cluster we select variables to represent each. We call a set of variables selected this way a *surrogate set*. We examine several strategies for constructing surrogate set; these fall into two categories: *degree*-based strategies, relying on node degree and *centrality*-based strategies, relying on node centrality.

From each network cluster, degree-based strategies select nodes according to degree, or the number of nearest-neighbuor nodes. A typical strategy is to select the node(s) with the highest degree, or *hub(s)*. Hubs are known to play important roles in networks, such as robustness and resilience in the face of breakdowns [43]. In networks of human brain function [18, 20, 44, 45], there is evidence that hubs play important roles [19, 21–23, 46]. Furthermore, in molecular genetics, hub genes selected from a co-expression network have been shown to be biologically-significant [47]. Let us denote this strategy, and the surrogate set it produces, as ***H***. In addition, we consider another degree-based strategy and surrogate set, ***HS***, which in addition to the cluster hubs select those nodes with the next-highest degree, which we call *subhubs*. Using these two strategies ***H*** and ***HS***, we select 66 and 159 variables, respectively, out of the 1373 variables forming our consensus network. For purposes of consistency, let us call this “strategy” of selecting all variables (except for a single outcome variable to be used in succeeding sections), as well as resulting set of variables, as ***A***, containing 1372 variables.

In contrast, centrality-based strategies select nodes according to their centrality within their respective clusters. A multitude of network centrality measures exist (with the node degree counted as one of them in some sources [48]); we use the intra-cluster *betweenness centrality* [49, 50] in this work. The betweenness centrality (BC) of a network node is based on the number of shortest paths in the network passing through the node, and in a sense is a generalization of the neighborhood criterion presupposed by the node degree measure. In its scaled form, it is given by Eq 3:

$$\begin{array}{c} BC=\frac{2}{\left(N-1)(N-2\right)}\sum_{j\neq i\neq k}\frac{\sigma_{jik}}{\sigma_{jk}} \end{array}$$ (3)

where *N* is the number of nodes in the network, *i* is the node being considered, *σ*_*jk*_ is the number of shortest-paths from *j* to *k*, and *σ*_*jik*_ is the number of shortest paths between *j* and *k* which pass through *i*. In calculating a node’s intra-cluster BC, only those nodes and edges belonging to the subnetwork given by that cluster are used, and *N* is replaced by the number of nodes in the cluster. Analogous to ***H***, let us call the selection strategy of choosing from each cluster the highest intra-cluster BC nodes (which we call *cores*), as well as its associated surrogate set, as ***C***. Similarly, in parallel to ***HS***, the strategy of choosing from each cluster the highest and next-highest intra-cluster BC nodes (cores and *subcores*) and its surrogate set we call ***CS***. As surrogate sets, ***C*** and ***CS*** contain 48 and 91 variables, respectively.

We then evaluate the performance of the four surrogate sets compared to that of ***A*** in successfully predicting the value of an index of successful aging, *SAGE*, described in a previous paper [5]. The variable *SAGE* rates a patient 1 based on fulfilling the following criteria, and 0 otherwise:

1. Absence of self–reported major diseases such as heart disease, diabetes, stroke, dementia, mental illness, chronic neurologic disease, end–stage renal disease and cancer (however, the presence of a cardiovascular risk factor such as hypertension or hypercholesterolaemia is tolerated)
2. Good to excellent self–rated health
3. Absence of disabilities on the Activities of Daily Living (ADL) and Instrumental Activities of Daily Living (IADL) scales
4. Mini–Mental State Examination (MMSE) score equal to or greater than 26 (out of 30)
5. Fewness or absence of depressive symptoms, corresponding to a Geriatric Depression Scale (GDS) score of less than 5
6. Good self–rated life satisfaction
7. High level of participation (at least once a week) in at least one social or productive activity, including social, recreational or civic activities

*SAGE* is thus a variable which takes into account the multi-faceted nature of aging, integrating physical, cognitive, and psychosocial aspects. In the consensus network shown by Fig 4, *SAGE* belongs to Cluster 22. However, it occupies a peripheral position within the cluster, having two nearest neighbors, and an intra-cluster BC of 0.0. Thus it is not selected by any strategy we considered above, and thus does not belong to any surrogate set or ***A***.

We use the surrogate sets and ***A*** as features to train machine learning models for each set. We split the SLAS-2 data set 75%-25% into a training set and a test set, and use the training set to perform 10-fold cross-validation for each model. As *SAGE* is a binary outcome variable, we plot receiver operating characteristic (ROC) curves for each model and use the area under the ROC curve (AUC) as the measure of performance of each selection strategy. A preliminary performance comparison between the gradient boosting machine (GBM), deep learning (DL) and distributed random forest (DRF) machine learning algorithms showed that for all five sets of variables, the GBM algorithm offered the best performance in predicting *SAGE*. Thus, we use it in the present study.

All models (both for the present study and the preliminary performance comparison) were trained on the H2O.ai platform [51, 52]. For each, a grid search across hyper-parameters was performed. Hyper-parameter values used in the grid search for each model type are listed in Table A in S1 File. For each combination of hyper-parameters and surrogate set, ten cross-validation models (one for each fold) were trained. The cross-validation model (with its hyper-parameter combination) with the best log-loss performance was selected as the model for that surrogate set, and used to predict *SAGE* for the test set.

## Results

### Consensus network properties

Measures of association (effect-size measures) and statistical significance tests were performed pairwise on the SLAS-2 dataset, and a network was constructed out of the set of pairwise effect-sizes that were significant at *α* = 0.05 after applying the Bonferroni correction. After obtaining the giant component of this network (Fig 1), a consensus network was formed by combining 250 minimum spanning trees (MSTs) of the giant component and applying the majority rule to the network edges. Fig 4a shows this consensus network. The network is not connected, being instead split into 7 components. The two smallest components each consist of a pair of connected nodes pertaining to a general health status score (*w0_eq5d4* and *EQ5D_score*) and activities of daily living (*W0_adl1c* and *ADLc*) unconnected to anything else. The biggest component (*N* = 1035, *E* = 1062), accounts for 75.38% of all the nodes and 76.18% of all the edges in the consensus network.

The nodes of the consensus network were clustered using the Louvain community detection method. Out of the 42 clusters obtained this way, the largest component accounts for 28 clusters; the second largest for 9, and the remaining five contain a single cluster each. Fig 4b shows a schematic diagram of the seven components and their clusters.

### Performance and benchmarking

**Fig 5:**
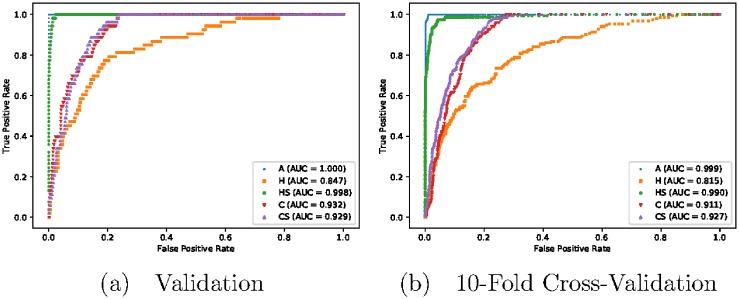
ROC curves for (a) validation and (b) 10-fold cross-validation of GBM classifiers trained on surrogate sets *H*, *HS*, *C* and *CS*. The ROC curves for ***A*** are also presented for comparison.

Four subsetting strategies were applied to the clusters obtained from the consensus network. Two were node degree-based (***H*** and ***HS***), and two were node centrality-based (***C*** and ***CS***). The full set of node-variables (except for the outcome variable *SAGE*, which additionally is absent from the four obtained subsets) was designated as ***A***, and used as a reference for the four subsets previously obtained (the *surrogate sets*). Gradient boosting machine (GBM) classifiers were trained on each of the five sets, with *SAGE* as the outcome variable. Fig 5 shows the receiver operating characteristic (ROC) curves for the classifiers trained on the four surrogate sets and ***A***, for both the validation set and 10-fold cross-validation. We use the area under the ROC curves (AUCs) as the comparison metric for the performance of the five. In the context of our work, this is because for a binary classifier, the AUC gives the probability that the model ranks a randomly-chosen subject with *SAGE = 1* higher than another subject, also randomly chosen, with *SAGE = 0*. An AUC of 0.5 then indicates that the model’s prediction performance is no better than chance, while 1.0 indicates perfect predictive performance. Models trained on *A* have AUC values equal to 1.0, or very close to it, since they use all information available in their construction. Those trained on the surrogate sets have lower AUCs due to working with less feature variables; we wish to examine these differences in performance in the context of the sizes of their surrogate sets. A good surrogate set is one that has minimal decrease in AUC relative to ***A***, while at the same time much smaller than the latter.

Among the surrogate sets, the degree-based ***H*** and ***HS*** have the worst and best predictive performance respectively, with the latter (AUC = 0.998 for validation, 0.990 for cross-validation) performing nearly as good as ***A*** (AUC = 1.000 for validation, 0.999 for cross-validation). ***C*** and ***CS*** perform similarly, with that of ***CS*** very slightly lower with the validation set (0.929 vs. 0.932), and slightly higher with the cross-validation (0.927 vs. 0.911). This indicates that, if one is to go for a centrality-based selection strategy, simply choosing the most central variables in each cluster (the *core* nodes) may be sufficient. Adding the next most central variables (the *subcores*) yields marginal or no improvement in predictive performance, at the cost of nearly doubling the number of variables used, 91 to 48. In contrast, while ***HS*** offered a significant improvement over ***H***, the size of the surrogate set is more than doubled, 159 to 66.

As a second means of benchmarking the surrogate sets, we create other subsets of ***A*** by randomly-choosing a fixed number *k* of variables from each cluster. Furthermore, for each subset created this way, we randomly sample an equal amount of variables from the entire set of 1373 variables. Sets obtained with and without cluster information (that is, sets created by randomly sampling each cluster and the whole variable set at large) are designated *StRS* (stratified random samples) and *SRS* (simple random samples) respectively. We then use the resulting subsets to train GBM models on *SAGE* (taking all the contained variables for clusters with sizes smaller than *k*).

**Fig 6:**
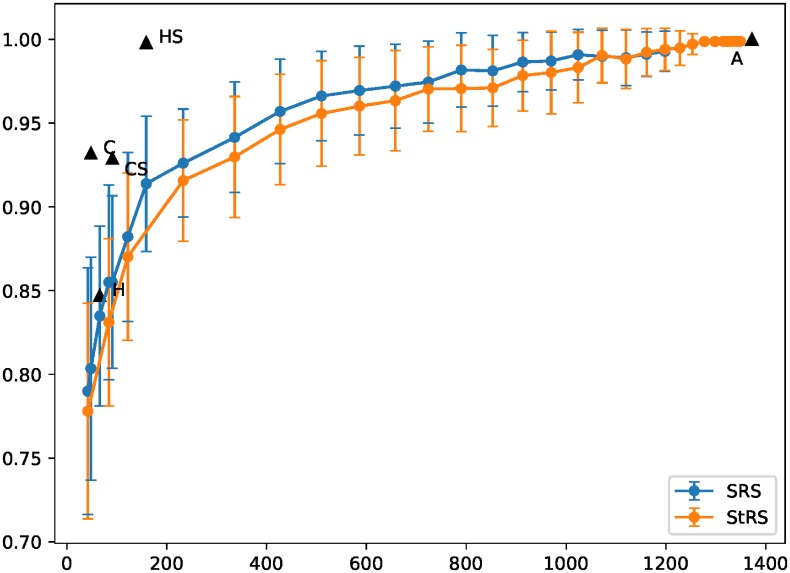
Performance comparison of GBM classifiers trained on *A* and surrogate sets *H*, *HS*, *C* and *CS* with classifiers trained on feature subsets formed by choosing *k* variables from the clusters defined by the majority-rule consensus network in Fig 4. All classifiers used the variable *SAGE* as the response. The horizontal axis is the size of the feature subsets and the surrogate sets, and the vertical axis is the area under the ROC curves (AUC). Values and error bars are averages and standard deviations of 100 replicates for each *k* for the randomly-selected features. Data points for the surrogate sets and **A** are AUC values for the validation set. The lines between data points are guides for the eye.

Fig 6 shows the average and standard deviation of the AUC (for both the validation set and cross-validation) from 100 replicates for each *k* versus the total number of variables of the sets corresponding to a given *k*. The validation set AUCs of ***H***, ***HS***, ***C***, ***CS*** and ***A*** are also shown along with their respective set sizes; the cross-validation AUCs, as given by Fig 5, are very close to their validation set counterparts and thus are not shown here.

We see that the predictive performance of ***H***, aside from being the lowest out of the four surrogate sets, seems to fall within the range expected of randomly-chosen subsets of ***A***, between the data points equivalent to *k* = 1 (one randomly-selected feature variable per cluster, 42 variables) and *k* = 2 (two feature variables per cluster, 84 variables). Those of the other three surrogate sets do not: ***C*** and ***CS*** both lie barely out the envelope created by the error bars, while ***HS*** lies further out.

### Relative importance of predictor variables

We examine the predictor variables in each surrogate set and ***A*** which contributed the most towards their corresponding models’ performance in predicting the outcome variable, using the *relative importance* (RI) scores for each variable in each GBM model. The RI of a predictor variable in a GBM model is a measure of how strongly the variable contributed to the quality of the model’s prediction. The GBM algorithm utilizes an ensemble of decision trees to make predictions, and for a single tree, a predictor’s RI is given by the number of times it was used in the tree splits, weighted by the reduction in the squared error resulting from each split. Friedman [53] gives the equation for the RI of a variable *j* in a single decision tree *T* with *L* splits as follows:

$$\begin{array}{c} {\hat{I}}_{j}^{2}\left(T\right)=\sum_{k=1}^{L-1}{\hat{i}}_{k}^{2}1\left(v_{k}=j\right) \end{array}$$ (4)

where *k* is a non-terminal split in *T*, *v*_*k*_ is its associated variable, ${\hat{i}}_{k}^{2}$ is the latter’s associated empirical reduction in the squared error, and 1 is the indicator function. The predictor’s RI was then averaged across the *N*_*trees*_ decision trees used by each GBM model, as given by the following equation:

$$\begin{array}{c} {\hat{I}}_{j}^{2}=\frac{1}{N_{trees}}\sum_{t=1}^{N_{trees}}{\hat{I}}_{j}^{2}\left(T_{t}\right) \end{array}$$ (5)

**Fig 7:**
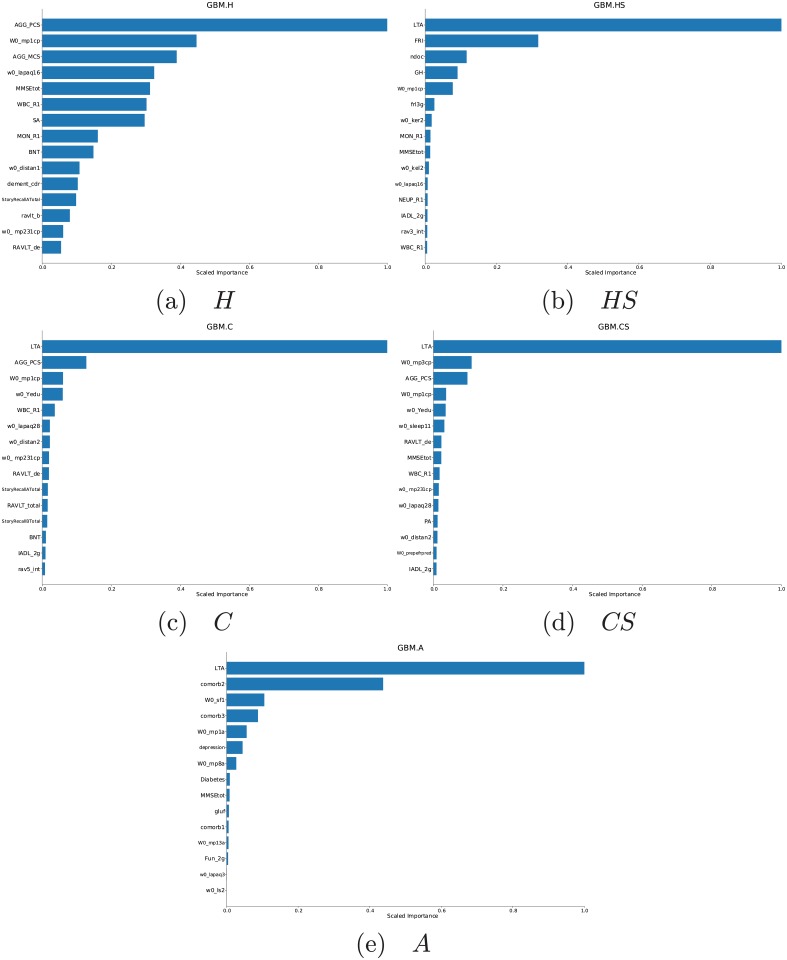
The 15 most important feature variables of GBM classifiers trained on the surrogate sets *H*, *HS*, *C*, *CS* and the full set *A*. The relative importances were scaled with respect to the most important feature variable for each. Full descriptions for each feature variable and surrogate set can be found from Table B to Table F in S1 File.

Fig 7 shows the fifteen most important predictor variables for each GBM model trained on the surrogate sets and ***A***. Here, the individual variable RIs have been given both unscaled, and scaled to that of the most important predictor for each model. The variables’ descriptions and corresponding RIs (scaled and unscaled) are contained in Tables B-F in S1 File. Each model’s top fifteen variables accounted for the bulk of its predictive performance: 91.6% for ***H***, 97.2% for ***HS***, 96.9% for ***C***, 94.3% for ***CS*** and 99.8% for ***A***. In the sets which have it, *LTA*, the subjects’ leisure-time activity score, accounted for more than half of the empirical squared-error reduction (from 55.4% for ***A*** to 67.1% for ***C***). Lower-ranked variables, from the 2nd down, each accounted for much smaller fractions of the squared-error reduction and thus individually are much less important than *LTA*.

In the exception (***H***), the highest-importance variable, *AGG_PCS*, is a subject’s physical health composite score according to the NEMC Short Form 12 Health Survey (SF-12) [54–56]. This variable only accounted for 23.5% of the squared-error reduction for the GBM model trained on ***H***. The lower-ranked variables get higher proportions, and thus have larger RI scores compared to their counterparts in the other sets. Without *LTA*, however, the average total squared-error reduction, obtained by summing all the unscaled RIs, for ***H*** (4962.99) is lower compared to the other three surrogate sets (***HS***: 5981.61; ***C***: 5213.86; ***CS***: 5290.17) and ***A*** (6137.77). Interestingly however, *LTA*’s social component *SA* (the most important contributor to *LTA* as determined from a coefficient analysis of its components) is the seventh-most important predictor of *SAGE* within ***H***.

## Discussion

In this paper, network clustering of clinical data from an elderly cohort was performed, with a view to obtaining subsets of variables (*surrogate sets*) which are representative of the whole. Surrogate sets obtained using network node degree-based and centrality-based strategies were evaluated for their performance in predicting an index of successful aging using machine learning classifiers. The choice of centrality-based surrogate sets (***C*** and ***CS***) to train GBM models offers increased predictive performance over similarly-sized sets of randomly-selected variables, whether clustering information was used (*StRS*) or not (*SRS*). The performance of models trained on degree-based surrogate sets was mixed: using hubs alone (***H***) did not yield performance different from those trained on randomly-chosen variables. Using subhub variables together with hubs (***HS***) yielded a marked improvement, with predictive performance very close to perfect prediction. This, however, comes at the cost of substantially increasing the size of the surrogate set. Random selection of variables does not consistently reach comparable performance until a very large proportion of the variables have been selected as training inputs; we see this regardless of whether clustering information was used or not.

The most important domains identifiable in each surrogate set are similar, with top-fifteen variables belonging to the physical, cardiovascular, cognitive and immunological domains captured by all four (and **A** as well). *LTA*, representing a fifth domain (leisure-time activities) is captured by all surrogate sets save ***H***; yet even here, the social score *SA* (the most important contributor to *LTA* within the cohort) is ranked seventh among the important variables. Medical-history domain variables are also absent among *H*’s most important variables, and those from the metabolic domain are present only in ***CS***, something also exhibited by ***A***.

While ***HS***, ***C***, and ***CS*** all perform above the envelope of randomly-selected variable sets, the centrality-based surrogate set ***C*** may offer a good balance between the set size and predictive accuracy compared to ***HS*** and ***CS*** (159 and 91 variables respectively, to ***C***’s 48). It is superior to ***H***, its degree-selection analog, in both set size and predictive performance. In common with the other surrogate sets, it covers several domains and thus can serve as a broad-based subset of the SLAS-2 data. In this context, we note that network centrality-derived measures have previously been used successfully to identify, *e.g*., key genes in gene expression data and proteins in protein-protein interaction networks [57–64], and in at least one instance have been used to identify breast cancer-related genes by contrasting between normal and tumor networks built from gene expression, mutation and protein-protein interaction data [65].

We offer some remarks regarding our network-and-clustering approach, and the comparative performance of centrality-based and degree-based subsetting strategies in particular. The consensus minimum-spanning network we obtained from its parent (a pairwise effect-size network) is effectively a network containing the strongest association strengths between pairs of variables (in a dataset with purely numerical variables, this strength is Spearman’s *r*^2^). The modularity maximization used by the clustering algorithm results in a partitioning in which intra(inter)-cluster associations are strongest(weakest). With this in mind, a degree-based approach picks those variables with the most number of strong *and* direct associations (neighbors) within their clusters, while a centrality-based approach picks those with strong associations, whether direct *or* indirect. The quasi-tree structure of the consensus network (obtained as it is from multiple minimum-spanning trees), combined with the large number of clusters we obtained, tends to make the intra-cluster betweenness centrality to drop off sharply past the most central variable within the cluster, which translates to negligible changes in prediction performance, something reflected by the comparative performance of ***C*** and ***CS***.

In general, the most central nodes and the highest-degree ones in the clusters do not coincide, and we see that in the case of *LTA*. By picking up variables which, by their position within their respective network clusters, “move together” the strongest with others (and thus are in positions which render the rest as redundant), a centrality-based strategy thus ensures a selection of a subset of variables representative of the fuller dataset.

While the variable stratifying the cohort used in this study (the *outcome variable*, in machine learning terms) is *SAGE*, the methods used to obtain our results are themselves data-agnostic; the input data, instead of being clinical variables, might be information derived from some other source (such as immunological assays, metabolomic or gene expression data), and the outcome variable of interest might be an indicator for the presence or absence of a specific medical condition (such as sarcopenia, diabetes mellitus or cardiovascular disease). In addition, the method is not restricted to categorical outcome variables; the GBM algorithm handles both categorical and numerical-valued outcome variables similarly. In the latter case, however, the AUC metric becomes inapplicable and an alternate performance metric, such as mean-squared error, may be substituted.

## Conclusion

In conclusion, the network-and-clustering based approach we described in this work yielded substantial reductions in the number of variables we obtained compared to the initial data set. Furthermore, the use of a centrality-based selection strategy yielded a good balance between the variable subset’s size and predictive performance with respect to a desired outcome variable. While we reported results specific to the SLAS-2 clinical dataset, the complete pipeline itself is input-agnostic and can be straightforwardly adapted to other data sources and desired exploratory and outcome variables. Further work will focus on clarifying the relationship between individual variables’ predictive performance (as given by their relative importances to a trained GBM model) and their position in the pairwise effect-size network, as well as a more general verification of whether the predictive performances of each surrogate set hold true across other networks, such as in gene regulatory and protein-protein interaction networks.

## Supporting information

S1 File Supporting information tables. (PDF)
