# Supplementary material for: Degree and centrality-based approaches in network-based variable selection: Insights from the Singapore Longitudinal Aging Study
Source: PLoS One. 2019 Jul 18;14(7):e0219186. doi: 10.1371/journal.pone.0219186 (PMC6638841; doi:10.1371/journal.pone.0219186)
Supplement: S1 File — (PDF) [file pone.0219186.s001.pdf]

Supporting information

Table A. Hyperparameters used in H2O.ai grid search.

Hyper-parameter	Description	Values
h_{max}	Maximum tree depth	5
λ	Learning rate	0.1
N_{bins}	Number of bins used in histogram	1024
N_{trees}	Number of trees built	50, 100, 150, 200
tol_{stop}	Stopping tolerance	0.001

Table B. The 15 most important features of a GBM classifier trained on *A*.

Variable	Description	Relative Importance	Scaled Importance
LTA	Leisure time activities (total score)	3403.2600	1.0000
comorb2	Total number of self-reported medical conditions (excluding depression and dementia)	1489.4579	0.4377
W0_sf1	Quality of Life: Self-evaluation of health	358.9681	0.1055
comorb3	Total number of all medical conditions (self-reports, medication, physical and laboratory tests)	299.0852	0.0879
W0_mp1a	Self-reported history of high blood pressure	191.2625	0.0562
depression	Depression (antidepressant use and self-reports)	152.5632	0.0448
W0_mp8a	Self-reported history of eye problems	92.4386	0.0272
Diabetes	diabetes (fasting glucose, self-reports and anti diabetic treatment)	30.9569	0.0091
MMSEtot	MMSE, total score	28.4086	0.0083
gluf	Fasting glucose (mmol/L)	22.4176	0.0066
comorb1	Total number of self-reported medical conditions	19.5800	0.0058
W0_mp13a	Self-reported history of arthritis	18.5383	0.0054
Fun_2g	Presence of any disability in ADL or IADL	14.9808	0.0044
w0_lapaq3	LASA Physical Activity: Number of times of walks in the past 2 weeks	2.8779	0.0008
w0_ls2	Life Satisfaction: Self-reported happiness in present life	1.4687	0.0004

Table C. The 15 most important features of a GBM classifier trained on *H*.

Variable	Description	Relative Importance	Scaled Importance
AGG_PCS	Physical health (NEMC) t-score – SF-12	1168.1864	1.0000
W0_mp1cp	Physician visits for high blood pressure	522.5864	0.4473
AGG_MCS	Mental health (NEMC) t-score – SF-12	455.5632	0.3900
w0_lapaq16	LASA Physical Activity: Sports that was done most frequently in the past 2 weeks	379.3896	0.3248
MMSEtot	MMSE, total score	365.1859	0.3126
WBC_R1	White blood cells ($\times 10^9$/L)	353.3155	0.3024
SA	Leisure time activities (social activity score)	346.9807	0.2970
MON_R1	Monocytes (absolute, $\times 10^9$/L)	188.6009	0.1614
BNT	BNT total	173.6624	0.1487
w0_distan1	Walking distance on a usual weekday (hours/day)	126.4335	0.1082
dement_cdr	Clinical dementia rating	120.6815	0.1033
StoryRecallATotal	Story Recall A total score	115.0009	0.0984
ravlt_b	RAVLT B recall score	93.8247	0.0803
w0_mp231cp	Health care provider visits for surgery procedure (1)	71.1444	0.0609
RAVLT_de	RAVLT delayed (existing variable)	64.0676	0.0548

Table D. The 15 most important features of a GBM classifier trained on *HS*.

Variable	Description	Relative Importance	Scaled Importance
LTA	Leisure time activities (total score)	3387.1780	1.0000
FRI	Frailty index (percentage of cumulative deficits)	1075.2416	0.3174
ndoc	Total number of physician visits in previous year	393.9638	0.1163
GH	General Health score (NEMC, US norms standardized)	308.2295	0.0910
W0_mp1cp	Physician visits for high blood pressure	262.7177	0.0776
frl3g	Frailty (phenotypic model, 3 categories)	87.5743	0.0259
w0_ker2	Knee extension, trial 2, right (kg)	61.5735	0.0182
MON_R1	Monocytes (absolute, $\times 10^9$/L)	49.8238	0.0147
MMSEtot	MMSE, total score	47.4154	0.0140
w0_kel2	Knee extension, trial 2, left (kg)	35.4213	0.0105
w0_lapaq16	LASA Physical Activity: Sports that was done most of the time in the past 2 weeks	23.6185	0.0070
NEUP_R1	Neutrophils (%)	23.0021	0.0068
IADL_2g	Presence/absence of IADL disabilities	21.5338	0.0064
rav3_int	RAVLT trial 3, intrusion errors	19.8732	0.0059
WBC_R1	White blood cells ($\times 10^9$/L)	18.6153	0.0055

Table E. The 15 most important features of a GBM classifier trained on *C*.

Variable	Description	Relative Importance	Scaled Importance
LTA	Leisure time activities (total score)	3497.6033	1.0000
AGG_PCS	Physical health (NEMC) t-score – SF-12	447.5523	0.1280
W0_mp1cp	Physician visits for high blood pressure	211.2916	0.0604
w0_Yedu	Years of schooling	209.0572	0.0598
WBC_R1	White blood cells ($\times 10^9$/L)	127.8283	0.0365
w0_lapaq28	LASA Physical Activity: Number of days in past 2 weeks when heavy household tasks were performed	78.2328	0.0224
w0_distan2	Walking distance on a usual weekend (hours/day)	78.1230	0.0223
w0_mp231cp	Health care provider visits for surgery procedure (1)	69.6459	0.0199
RAVLT_de	RAVLT delayed score	68.3553	0.0195
StoryRecallATotal	Story Recall A total score	57.1774	0.0163
RAVLT_total	RAVLT total score	55.9418	0.0160
StoryRecallBTotal	Story Recall B total score	51.5331	0.0147
BNT	Boston Naming Test (total score)	37.5895	0.0107
IADL_2g	Presence/absence of IADL disabilities	33.9414	0.0097
rav5_int	RAVLT trial 5 intrusion errors	28.4251	0.0081

Table F. The 15 most important features of a GBM classifier trained on *CS*.

Variable	Description	Relative Importance	Scaled Importance
LTA	Leisure time activities (total score)	3446.0029	1.0000
W0_mp3cp	Physician visits for diabetes	378.7017	0.1099
AGG_PCS	Physical health (NEMC) t-score – SF-12	337.4351	0.0979
W0_mp1cp	Physician visits for high blood pressure	126.5989	0.0367
w0_Yedu	Years of schooling	121.9288	0.0354
w0_sleep11	Sleep: frequency of having trouble sleeping in past month due to other reasons	108.9992	0.0316
RAVLT_de	RAVLT delayed (existing variable)	79.5969	0.0231
MMSEtot	MMSE, total score	78.5525	0.0228
WBC_R1	White blood cells ($\times 10^9$/L)	62.0728	0.0180
w0_mp231cp	Health care provider visits for surgery procedure (1)	53.9533	0.0157
w0_lapaq28	LASA Physical Activity: Number of days in past 2 weeks when heavy household tasks were performed	49.9279	0.0145
PA	Leisure time activities (physical activity score)	y 42.1722	0.0122
w0_distan2	Walking distance on a usual weekend (hours/day)	40.9136	0.0119
W0_prepefrpred	Peak expiratory flow rate (pre-bronchodilator), % predicted	32.0349	0.0093
IADL_2g	Presence/absence of IADL disabilities	31.4441	0.0091
